# Development and Clinical Evaluation of a Multiplexed Health Surveillance Panel Using Ultra High‐Throughput PRM‐MS in an Inflammatory Bowel Disease Cohort

**DOI:** 10.1002/anie.202507610

**Published:** 2025-09-22

**Authors:** Qin Fu, Philip M. Remes, Jihyeon Lee, Cristina Jacob, Dalin Li, Manasa Vegesna, Koen Raedschelders, Ali Haghani, Emebet Mengesha, Philip Debbas, Esthelle Hoedt, Sandy Joung, Susan Cheng, Scott Peterman, Justyna Fert‐Bober, Gil Y. Melmed, Dermot P.B. McGovern, Christopher I. Murray, Jennifer E. Van Eyk

**Affiliations:** ^1^ Cedars‐Sinai Medical Center Los Angeles CA USA; ^2^ Thermo Fisher Scientific San Jose CA USA; ^3^ Present address: Thermo Fisher Scientific San Jose CA USA; ^4^ Present address: Ultragenyx Novato CA USA

**Keywords:** Clinical biomarker translation, Inflammatory bowel disease, Targeted peptides, Validation proteomics

## Abstract

Despite advances in clinical proteomics, translating protein biomarker discoveries into clinical use remains challenging due to the technical complexity of the validation process. Targeted MS‐based proteomic approaches such as parallel reaction monitoring (PRM) offer sensitive and specific assays for biomarker translation. In this study, we developed a multiplex PRM assay using the Stellar mass spectrometry platform to quantify 57 plasma proteins, including 24 FDA‐approved biomarkers. Loading curves (11 points) were performed at 4 sample throughputs (100, 144, 180, and 300 samples per day) using independently optimized and scheduled PRM methods. Following optimization, an inflammatory bowel disease (IBD) cohort of plasma samples (493 IBD, 509 matched controls) was analyzed at a throughput of 180 samples per day. To monitor system performance, the study also included over 1000 additional injections for system suitability tests, low‐, middle‐, and high‐quality controls, washes, and blanks. Using this approach, we observed high quantifiability (linearity, sensitivity, and reproducibility) in the PRM assay and consistent data acquisition across a large cohort. We also validated the candidate IBD markers, C‐reactive protein and orosomucoid protein, identified in a recent discovery experiment.

## Graphical abstract

Validating biomarkers of disease is necessary for clinical use. The Health Surveillance Panel (83 peptides from 57 proteins) was developed as a PRM assay and evaluated using the Stellar‐MS platform. The assay was validated with a large clinical cohort of inflammatory bowel disease patients alongside quality controls. Analysis demonstrates strong performance, disease insight, and a preferred approach for future high‐throughput biomarker validation.

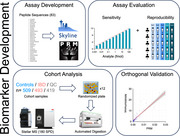

## Introduction

Blood plasma is a readily accessible biofluid that reflects the physiological state of an individual, making it an invaluable source for protein biomarkers of disease.^[^ ^1^ ^]^ Once a potential biomarker has been identified in an initial discovery experiment, the clinical utility must be rigorously validated in large patient cohorts. One widely used approach for biomarker validation is a mass spectrometry (MS)‐based targeted protein assay, where a peptide (precursor) that uniquely represents the candidate protein is quantified by measuring its intensity across the chromatographic peak during elution. Each targeted assay must be precise, reliable, and robust across multiple runs and scalable to accommodate large sample sets typical of clinical studies.^[^ ^2^, ^3^, ^4^, ^5^ ^]^ An advantage of a targeted MS protein assay is the ability to multiplex analytes and measure several candidate biomarkers in a single acquisition. This efficiency improves sample throughput and reduces instrument time when analyzing a large validation cohort. However, building a high‐quality targeted multiplex assay remains challenging, as each peptide analyte must be optimized and scheduled with respect to the others, which can be resource‐intensive and time‐consuming. Currently, there is no single solution to address the scale, sensitivity, and throughput demands of a multiplexed assay that can meet the rigor of clinical validation.

Several targeted MS‐based techniques, including multiple reaction monitoring (MRM) and parallel reaction monitoring (PRM), have been successfully deployed to validate candidate biomarkers.^[^ ^6^, ^7^ ^]^ MRM, typically performed on triple quadrupole instruments, has become a mainstay for quantitative protein assays in clinical laboratories.^[^ ^8^, ^9^ ^]^ In an MRM assay, the desired precursor is isolated in the first quadrupole (Q1), fragmented in the second (Q2) via collision‐induced dissociation and verified by monitoring multiple predefined fragment ions, also referred to as transitions, in the third quadrupole (Q3). The MRM triple quadrupole approach excels at targeted detection; however, it requires defining MRM transitions pre‐experiment, since triples are not well‐suited for acquisitions that scan over a wide *m*/*z* range.^[^ ^10^ ^]^ PRM assays differ in that all fragment ions from a targeted precursor are accumulated in parallel and measured in a single acquisition event. The parallel accumulation gives PRM a sensitivity advantage over MRM; it also greatly simplifies the experimental definition and effectively increases selectivity because transitions can be defined post‐experiment. PRM assays have traditionally been performed on quadrupole‐orbitrap or quadrupole‐time of flight (TOF) mass spectrometry systems. Quadrupole‐TOF systems are fast, and the latest generation is sensitive, while generally offering lower resolution and mass accuracy compared to quadrupole‐orbitrap mass spectrometers. Quadrupole‐orbitrap mass spectrometers conversely, provide higher resolution and mass accuracy but have lower acquisition rates and use a less sensitive image‐charge detection mode compared to the faster triple quadrupole and TOF instruments, which use electron‐multiplier‐based detectors.^[^ ^11^, ^12^ ^]^ A recent advancement has introduced a dual‐pressure linear ion trap in the Q3 position of the triple quadrupole design.^[^ ^13^ ^]^ Replacing the third quadrupole with a linear ion trap creates a hybrid instrument with triple quadrupole‐like speed and the PRM sensitivity advantage, albeit with unit mass resolution and accuracy. This new instrument configuration allows for improved acquisition rate (up to 140 Hz), greater ion storage, higher mass resolution, faster cycle time, extended dynamic range, and MS*^n^* acquisition.^[^ ^13^ ^]^ This MS platform also uses the Thermo Scientific adaptive RT algorithm to adjust acquisition windows in real‐time to account for retention time (RT) drift and the Skyline‐based tool, PRM conductor, that automates the selection and dynamic scheduling of precursors, simplifying the assay development process.^[^ ^13^ ^]^

To test the hybrid configuration, we re‐developed our previously reported Health Surveillance Panel (HSP) MRM multiplex assay into a multiplexed PRM format.^[^ ^14^, ^15^ ^]^ The HSP consists of 83 peptides representing 57 proteins and was originally designed as a diagnostic tool to measure a slate of laboratory‐developed tests (LDT) biomarkers related to cardiovascular, inflammatory, and metabolic conditions. The assay panel leverages 24 protein biomarkers that are already FDA‐approved and widely used in clinical practice (Table S1). These include C‐reactive protein (CRP) and orosomucoid protein (A1AG1),^[^ ^16^, ^17^ ^]^ well‐established inflammatory markers frequently measured to monitor activity, severity of flare, or response to therapy in inflammatory bowel disease (IBD) with some assays, such as those for CRP, already implemented for routine use in disease monitoring, including IBD.^[^ ^18^, ^19^, ^20^ ^]^ In the current study, we tested the HSP PRM assay using an 11‐point dilution series at four different sample throughputs to find the optimal balance in speed and quantification of all proteins. Next, we applied our optimized PRM assay to plasma samples from over 1000 individuals, including IBD cases and controls. IBD, comprising Crohn's disease (CD) and ulcerative colitis (UC), is a complex immune‐mediated disorder influenced by genetic predispositions and environmental factors.^[^ ^21^ ^]^ While the mechanisms underlying IBD pathogenesis and progression have been extensively studied, significant gaps remain in understanding the drivers of disease variability and therapeutic responses.

While endoscopic evaluation remains the gold standard for diagnosis, treatment assessment, and postoperative surveillance for IBD, this approach is invasive, costly, and not always feasible, especially for long‐term or continuous assessment. Biomarkers such as circulating CRP^[^ ^22^, ^23^, ^24^ ^]^ and fecal calprotectin (FCP)^[^ ^25^ ^]^ are the most widely used noninvasive alternatives in clinical practice.^[^ ^26^ ^]^ CRP is particularly useful for assessing moderate to severe disease and detecting complications. However, its sensitivity varies depending on disease type, location, and severity, and it may be normal in patients with mild or localized disease. Therefore, CRP should be interpreted alongside clinical evaluation and complementary biomarkers such as FCP and others.^[^ ^27^, ^28^ ^]^ Using the new hybrid MS platform, we validate candidate protein biomarkers and assess the performance of a large‐scale multiplex PRM assay in over 1000 patient samples. Together, these findings demonstrate the feasibility of large‐scale, multiplexed PRM assays for clinical biomarker validation and lay the foundation for broader implementation of translational proteomics and precision medicine research.

## Results and Discussion

### PRM Method Development

**Figure 1:**
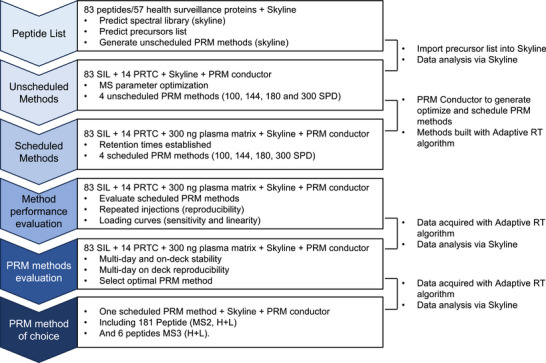
Schema of scheduled PRM methods development, evaluation, and establishment of preferred method for large scale and high‐throughput analysis.

We evaluated a modified 57‐protein multiplexed HSP PRM method on a high‐speed hybrid nominal mass platform (Stellar‐MS, Thermo Fisher Scientific). This HSP PRM method was developed based on a previously published MRM assay.^[^ ^14^, ^29^ ^]^ The current iteration of the assay consists of 83 prototypic peptides, their corresponding SIL peptides, and a 14‐peptide retention time calibration (PRTC) mixture (Thermo Fisher Scientific, Rockford, IL), totaling 180 peptides. To develop a large‐scale PRM assay, we assessed four different throughputs: 100, 144, 180, and 300 samples per day (SPD) to minimize acquisition time. Method development is a multistep process, as illustrated in Figure 1. Methods for each sample throughput were developed using Skyline with Prosit to predict the spectral library, precursors, and indexed retention times (iRTs) necessary to generate an unscheduled PRM method for the 83 different peptide sequences.^[^ ^30^, ^31^, ^32^, ^33^ ^]^ Instrument parameters for each unscheduled method were optimized using PRM conductor and Skyline using the results from replicate injections of the 83 SIL peptides (120 fmol) and with the PRTC mixture (50 fmol) spiked into 300 ng of digested plasma.^[^ ^13^ ^]^ SIL and PRTC concentrations are higher than those used for the remainder of the study to ensure observation while establishing the assay parameters. Detailed methods can be found in the Supporting Information, including throughput‐dependent PRM conductor parameters (Table S2) and the LC conditions, peptide retention times, precursor *m*/*z*, and charge states (Tables S3 and S4).

### Evaluation of Four Throughput Methods for Reproducibility, Sensitivity, and Linearity

**Figure 2:**
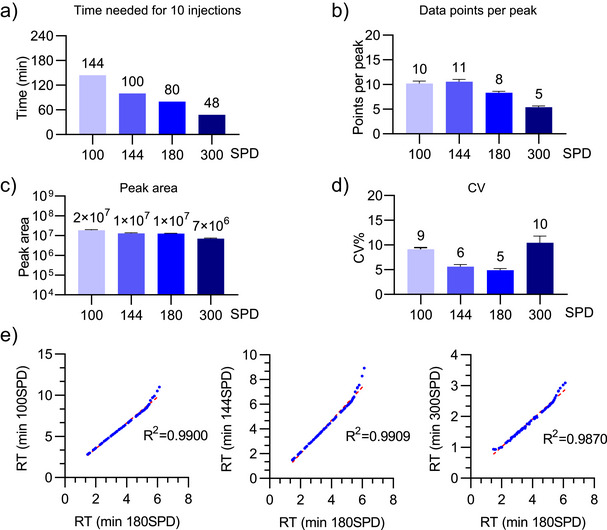
Technical evaluation of PRM methods with repeated injections. The performance of the four SPD methods was evaluated using 30 fmol of the 83 SIL peptides spiked into 300 ng of digested control plasma (*n* = 10 technical). a) Indicates the time required for 10 injections with each method. b) The median cross‐peak points. c) The median peak intensities. d) The median coefficient of variation (CV) of the signal intensities. e) Correlation between the retention times of 83 SIL across any two throughput conditions.

The performance of the four different throughput methods (100, 144, 180, and 300 SPD) was evaluated using an 11‐point SIL peptide dilution curve spiked into a pooled human plasma sample from 100 healthy females and 100 healthy males. As a benchmark, the time for 10 injections is shown for each throughput method (Figure 2a). For each dilution curve, points per peak, peak area, and coefficient of variation (CV) were assessed for all 83 peptides (Figure 2b–d). As expected, the median number of points across each precursor peak decreased with increasing sample throughput. Only the 300 SPD method failed to meet the required 7 points per peak for accurate quantification.^[^ ^34^ ^]^ Furthermore, the median peak areas also decreased with increasing sample throughput. The median CV values were all ≤10%, indicating good reproducibility across all four methods.

A key aspect of developing a multiplexed PRM assay is consistency in retention time to conduct effective scheduling of multiple precursors. We examined the retention time correlation across all four throughput settings for 83 SIL. Pairwise correlation analysis between any two throughput settings demonstrated excellent correlation, with *R* ^2^ values exceeding 0.98 (Figure 2e).

**Figure 3:**
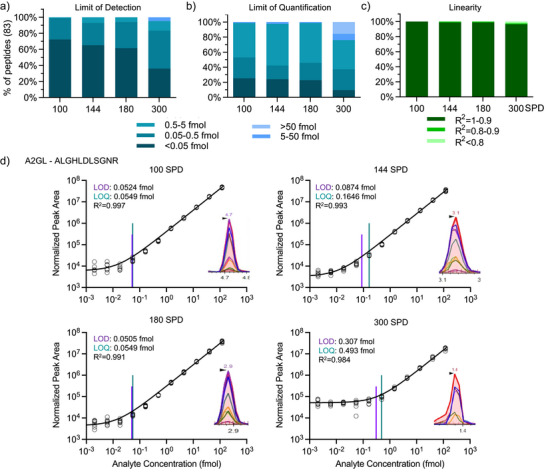
Evaluation of the sensitivity and linearity of each peptide in the PRM assay for the different throughput methods. 0.002–120 fmol 83 SIL/300 ng pooled plasma peptides was evaluated for each SPD method (11‐point dilution curve; *n* = 5 technical). The LLOD a) and LLOQ b) was calculated using the Skyline bilinear regression fit. The results are binned into five ranges as indicated. c) Median linear response (R^2^) for all 83 SIL peptides over the dilution curve. d) Examples of linear regression curves for A2GL peptide ALGHLDLSGNR are presented for 100, 144, 180, and 300 SPD. The peaks displayed are from Skyline showing integrated peaks of the same peptides. LLOD/Q data for all peptides and throughputs can be found in Table S5.

Next, the sensitivity, linearity, lower limit of detection (LLOD), lower limit of quantification (LLOQ), and reproducibility for each of the 83 peptides were determined across the 11‐point dilution curves for each SPD method (Figure 3). The observed median sensitivity for the 83 SIL peptides across all the methods was sub‐fmol (LLOD < 0.5 fmol), with the longer SPD methods achieving a higher percentage of peptides at or below this level (Figure 3a). The performance of the LLOQ (<5 fmol) was similar among 100, 144, and 180 SPD. There was a significant drop‐off at the 300 SPD throughput (81%–82% for the lower SPDs versus 62% for 300 SPD) (Figure 3b). The linear response of each peptide was also assessed, indicating that more than 95% of all peptides demonstrated an *R* ^2^ > 0.9 across the 11‐point dilution curve (Figure 3c) for all the SPD methods. Using the A2GL_ALGHLDLSGNR SIL peptide as an example, Figure 3d shows SIL standard curves across four different SPDs, along with LLOD, LLOQ, *R* ^2^ values, and precursor chromatographs. The asymmetric and imperfect chromatograms observed for this peptide at 300 SPD are consistent with low data points per peak and a relatively high LLOQ. The LLOQ, LLOD, and *R* ^2^ for each peptide at all four SPDs are summarized in Table S5. Based on the initial characterization of the methods, the 180 SPD method was found to have the preferred balance of performance and sample throughput. All subsequent evaluations were performed using this method.

### PRM Assay Reproducibility

**Figure 4:**
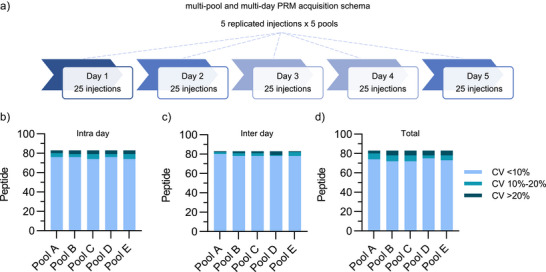
Evaluation of PRM assay technical reproducibility. a) Schematic outlining the analysis of five unique pooled plasma samples collected over 5 different days using the 180 SPD method (*n* = 5 biological; *n* = 5 technical). Each plasma pool was created by combining eight individual plasma samples; a total of 40 samples used to generate five distinct plasma pools. Summary of the median intra‐day b) and inter‐day c) CV for all 83 SIL peptides. d) Total variability was assessed using the mean intra‐day CV (across 5 days) and the mean inter‐day CV (across all five replicates) with the following formula: CV total​ = (CV intra^2^​ + CV inter^2^​)^1/2^. The total median variability is reported for each pool. Reliability data for each peptide is presented in Table S6.

The precision of the PRM assay on the Stellar MS platform was assessed for within‐sample and across‐day repeatability using a 5 × 5 × 5 experiment.^[^ ^35^ ^]^ Five injections per day over 5 days were performed using a number of independently pooled human plasma samples (labeled as Pools A–E, with each pool consisting of eight different plasma samples) spiked with the 83 SIL peptide panel (120 fmol) and the PRTC mixture (50 fmol) (Figure 4a). The mean intra‐assay (same day, between pools), inter‐assay (same pool, between days), and total (all pools, all days) coefficients of variation (CV) were calculated from the area ratio of the endogenous and SIL peptides (light/heavy ratio) (Figure 4b–d) (see Table S6). For the intra‐day precision of the 83 peptides in the 5 different plasma pools tested, 74 (C and E) or 76 (A, B, and D) of the peptides had intra‐day mean CV of <10%, with only 3 (A) or 4 (B–E) peptides >20%. The evaluation of between‐day precision revealed that 78 (B–D) or 80 (A) peptides had inter‐day mean CV of <10% with 1 (A and E), 2 (B and C) or 4 (D) >20%. Finally, across all 125 injections spanning the 5 days, 72 (B and C), 73 (A and E), or 74 (D) peptides had total experiment CVs of <10% and only 3 (A) or 5 (B–E) peptides had a CV >20%. All reproducibility data is presented in Table S6. The peptides observed with greater than 20% CV inter‐, intra, or total were due to low concentration of the native peptide in the plasma or an inherent high variability in MS ionization leading to inconsistent measurements. Analysis of the heavy peptides indicated two peptides, SHBG_VVLSQGSK and CRP_ESDTSYVSLK, were not reliably detected with average total CVs <70%.

### PRM Data Acquisition of the IBD Patient Cohort

**Figure 5:**
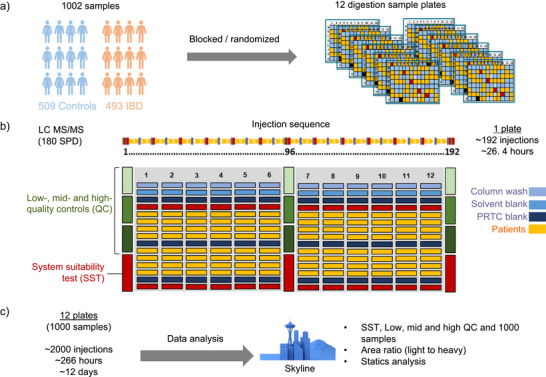
Design of 57‐Plex PRM assay for patient cohort analysis. a) Schema for processing and assignment of randomized blocks of subject plasma samples in 12 96‐well plates for analysis. b) Schematic of the plate layout for the patient and QC samples. Each plate included LC column washes, solvent banks, and PRTC blanks for every eight IBD cohort samples. System suitability tests (SST) were monitored for every eight samples. Quality controls were implemented at three levels: high, mid, and low (100, 20, or 5 fmol SIL/300 ng plasma), assessed at the beginning, middle, and end of each plate. The complete acquisition process for 1002 samples (12 plates) took over 12 days and >2000 injections. c) PRM peak integration and data analysis for the SST, QC, and patient samples were performed using Skyline.

As a proof of principle for the platform, we evaluated the application of the HSP 57‐protein multiplex PRM assay in a clinical cohort. We assembled an IBD cohort that comprised 1002 individual plasma samples (*n* = 493 IBD patients and *n* = 509 age‐ and sex‐matched healthy control subjects). Ethical approval to obtain human samples and perform the research outlined was obtained from the Institutional Review Board of Cedars‐Sinai Medical Center on Research Involving Human Subjects (Cedars‐Sinai Medical Center IRB#: Study 00001411 and Study 00000621). The study was conducted in accordance with the ethical principles specified in the Declaration of Helsinki. Informed consent was obtained from all participants. The full cohort was processed across twelve 96‐well plates using an i7 automated workstation (Beckman Coulter). The i7 automation workstation was previously set up for routine bottom‐up sample preparation, which includes protein denaturation, Cys reduction and alkylation, trypsin digestion, and desalting.^[^ ^36^ ^]^ After desalting, the 83 SIL (30 fmol) and 14 PRTC (5 fmol) peptides were spiked in prior to LC‐MS PRM analysis. The amount of reference standard was selected based on prior optimization with an emphasis on limiting overall variability across the panel and estimated clinical concentration of each analyte. LC‐MS acquisition was completed over 12 days at 180 SPD. The PRM acquisition sequence schema was designed to maximize instrument utilization by continuously acquiring data while simultaneously evaluating LC‐MS system performance. Throughout a 96‐well sample plate, plasma sample injections were intermixed with an equal number of injections for quality assurance (Figure 5). Column washes and blank solvent injections were performed every eight samples to assess column carryover. Randomized patient samples were run in blocks of 4 with a PRTC blank acquired before and after each block. Before and after every two‐patient block, a system suitability test (SST) was performed. The SST samples comprising 300 ng pooled plasma peptides spiked with 83 SIL (30 fmol) and 14 PRTC (5 fmol) peptides were used to assess drift in retention time. Finally, at the beginning, midpoint, and end of each plate, three quality control (QC) samples were run, with 300 ng of control plasma spiked with 14 PRTC (5 fmol) and 83 SIL peptides at high (100 fmol), middle (20 fmol), and low (5 fmol) concentrations to monitor signal stability and instrument performance.

**Figure 6:**
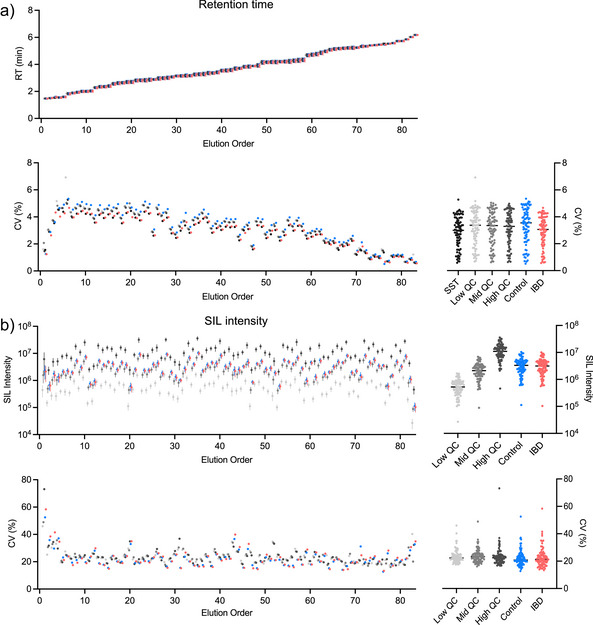
Assessment of PRM assay for patient cohort acquisition. a) Plot of the mean RT (above) and %CV (below) for the SST (black), QC (greys), control (blue), and IBD (red) samples for the 83 SIL peptides (error bars = SD; *n* = 311 SST; *n* = 36 QCs; *n* = 509 Control; *n* = 493 IBD). The peptides are ordered by column elution. Side panel shows distribution of CVs for each sample type. b) Plot of the mean SIL intensity (above) and CV (below) for the QC, control, and IBD samples. Side panels show distribution of SIL intensities and CVs for each sample type.

In total, 311 SST runs were completed during the acquisition of the entire 1002‐sample IBD cohort dataset. The RT of the 83 spiked SIL peptides demonstrated good stability. On average, the first peptide (SHBG_VVLSQGSK) eluted at 1.474 ±0.015 min, whereas the last peptide (LUM_SLEYLDLSFNQIAR) eluted at 6.177 ±0.039 min (Figure 6a). The median CV for the retention times was 3%, indicating strong stability across all 83 peptides. A similar RT performance was observed for the QC and patient samples. The total peak area of the 83 heavy peptides had CVs of 21%–23% in the QCs and patient samples, indicating the strong reproducibility of the SIL signal throughout the data acquisition process (Figure 6b). The evaluation of the QC samples showed good consistency across the cohorts. Of note, while the first and last peptides had stable RT, they did have poorer than anticipated performance. SHBG_VVLSQGSK (peptide 1, RT = 1.47 min) SIL intensity had CVs between 45% and 73% in the QC samples, which is consistent with its poor performance in the reliability study. Hydrophilic peptides tend to elute earlier and may not be sufficiently retained on the C18 column, making the measurement of these earliest four peptides by MS more variable or inconsistent.^[^ ^37^, ^38^ ^]^ This level of technical inconsistency suggests that SHBG_VVLSQGSK is unlikely to be usable for subsequent cohort analysis. The latest eluting peptide, LUM_SLEYLDLSFNQIAR (peptide 83, RT = 6.18 min), also had a higher SIL CV and displayed lower than expected SIL intensity (Figure 6b). Given the late elution, we examined the gradient and elution time (shown in Table S3, LC conditions). A possible reason could be that for 180 SPD, the entire LC time is 6.9 min and at 5.8 min, the gradient went into the washing phase, ramping the acetonitrile from 35% B to 99% B in 0.5 min with an increase in flow rate. Therefore, the last peptides were likely eluted more rapidly and with higher organics. For future experiments, the LC gradient will be optimized for early and late eluting peptides to achieve good signal reproducibility. Detailed characterization of the SIL intensity is presented in Table S7.

**Figure 7:**
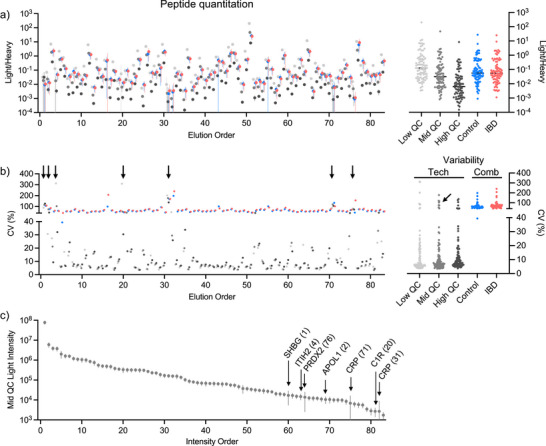
Evaluation of technical and biological variability in PRM assay quantitation for QC and patient cohort acquisition. Plot of the mean ratio of light to heavy peptide intensities a) and CVs b) for each of the SIL peptides (error bars = SD; *n* = 36 QCs; *n* = 509 Control; *n* = 493 IBD). The peptides are arranged by order of their column elution. Side panels show distribution of ratios and CVs by sample type. Technical (Tech) variability (QCs CVs) is lower than combined (Comb) technical and biological variability in the patient samples for all but seven peptides. Arrows in b) indicate seven peptides with CV > 40% in the QC samples. c) Plot of the mean light peptide intensity for the mid QC samples (error bars = SD; *n* = 36). Peptides are ordered by descending intensity. The seven peptides indicated in panel b) are labeled along with their column elution position.

Next, we evaluated the quantitative performance of the assay using the area ratio of the endogenous and SIL peptides. Across the elution profile, many peptides were quantified reliably in the QC samples, with median CVs of 7%–9% (Figure 7a,b). The technical variability was found to be much lower than the combined technical–biological variability observed in patient samples (Figure 7b). We observed seven peptides in the panel (indicated by arrows in Figure 7b,c) that had CVs > 40% and exceeded the observed variability in the patient samples. The poor performers are among the lower‐intensity peptides in the panel making them more susceptible to matrix interference in effectively isolating the peptide.

For most regulatory and CLIA‐compliant clinical assays, coefficients of variation (CVs) well below 20%, often ≤10%, are expected at clinically actionable concentrations. However, in research‐focused and developmental PRM workflows, particularly when targeting challenging or low‐abundance peptides, CVs up to 20%–25% are still commonly accepted.^[^ ^39^, ^40^, ^41^, ^42^ ^]^ In our analysis, we evaluated QC runs at multiple concentrations in parallel with a large clinical cohort. Figure 7b highlights the contrast between technical variability (derived from QC samples) and biological variability (derived from patient samples), with patient samples having >100% CV for nearly all peptides. The technical CV distribution falls into the following categories: 61% (<10%), 20% (10%–20%), 5% (20%–25%), and 14% (>25%) demonstrating the majority of the PRM panel is consistent with acceptable limits for reliability.

### LC‐MS^3^ Strategy Increases the Sensitivity and Specificity

**Figure 8:**
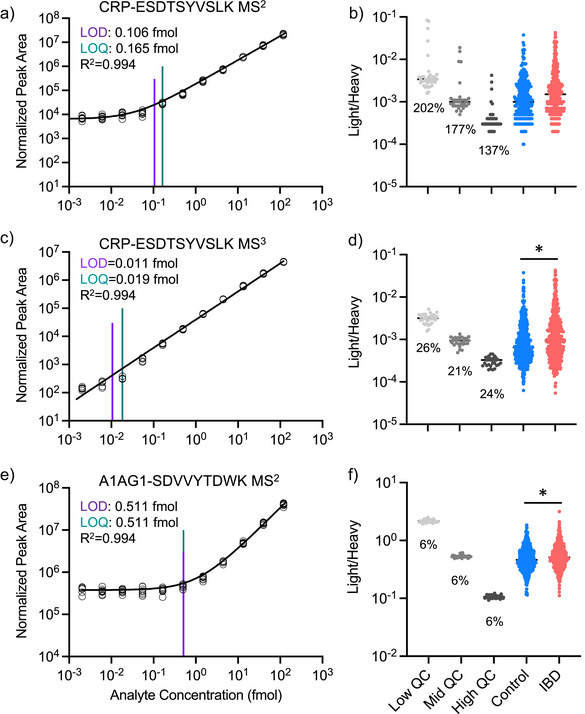
Detection and validation of candidate IBD biomarkers. PRM measurement of CRP precursor ESDTSYVSLK over the dilution series (11‐points) using MS^2^ a) and MS^3^ c) detection to measure LOD (purple) and LOQ (teal) for each approach (*n* = 5 technical). Panels to the right show summary of the light to heavy peptide ratios for MS^2^ b) and MS^3^ d) in the QC samples and patient cohorts. CV values for the QC samples are listed below points. The MS^3^ approach increased sensitivity and reduced technical variability for CRP measurements. CRP was significantly increased in IBD patients compared to controls (*n* = 509 control; *n* = 493 IBD; *P* = 0.0001) e) PRM detection of A1AG1 precursor SDVVYTDWK in MS^2^ mode over the dilution series (11‐point) to measure LOD and LOQ (*n* = 5 technical). f) Summary of A1AG1 QC and patient cohort analysis. A1AG1 was significantly increased in IBD patients (*n* = 509 control; *n* = 493 IBD; *P* = 0.00625).

One option to potentially increase selectivity, sensitivity, and reliability is the use of MS^3^ to further isolate, fragment, and detect product ions.^[^ ^43^ ^]^ This can reduce interfering ions that may obscure the peptide of interest. While MS^3^ acquisition potentially offers higher selectivity and lower limits of quantification, especially useful in complex backgrounds, this needs to be balanced against potential lower sample throughput, reduced spectral acquisition speed, and fewer ions per spectrum than MS^2^. A further consideration is that method development and optimization can be more involved for MS^3^ acquisition. We compared the MS^2^ and MS^3^ strategies for the CRP peptide (CRP_ESDTSYVSLK) (Figure 8a–d). In the 11‐point dilution curve, we observed that the targeted MS^3^ method demonstrated a nine‐fold increase in sensitivity, with an LLOQ of 0.0183 fmol mL^−1^ for MS^3^ compared with 0.1646 fmol mL^−1^ for MS^2^. The CRP MS^3^ method was subsequently incorporated into the final HSP PRM assay method. Evaluation of cohort QC samples also found a dramatic reduction in CVs from an average of 172% to 23%, putting CRP in line with the other well‐performing low‐abundance peptides in the panel. The 9‐ and 7‐fold improvements in sensitivity and reliability highlight the platform's acquisitional flexibility to measure low‐abundance peptides effectively. Furthermore, by targeting specific ion fragments, MS^3^ minimizes interference from co‐eluting or isobaric peptides, which is crucial for complex matrices like plasma or serum. Further testing is necessary to determine if an MS^3^ approach could improve the detection of the other poorly performing peptides in the panel.

### Validation and Discovery of IBD Biomarkers Using the HSP PRM Assay

In our previously published IBD discovery research, CRP and A1AG1 were identified as significantly upregulated proteins in the IBD population.^[^ ^17^ ^]^ CRP is routinely measured as part of the diagnostic and monitoring procedures for IBD patients^[^ ^44^ ^]^ and A1AG1 has been associated with IBD.^[^ ^16^ ^]^ In our targeted PRM study, both CRP_ESDTSYVSLK and A1AG1_SDVVYTDWK are included in our 57 protein‐HSP panel. Consistent with the discovery results, our PRM analysis with internal standard analysis validated the CRP and A1AG1 results, both were found to be upregulated in the IBD population (*P* = 0.0001 for CRP and *P* = 0.00625 for A1AG1) (Figure 8).

**Figure 9:**
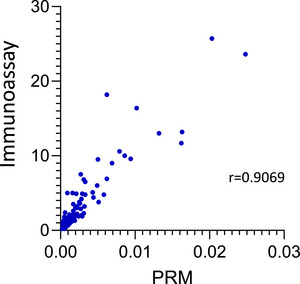
Comparison of PRM with clinical immunoassay measurements for CRP. Plot of the correlation of CRP PRM‐MS based analysis (area ratio of light/heavy) and immuno‐based assay (ng mL^−1^) in IBD patient plasma (*n* = 91). A Spearman correlation test revealed a strong correlation between the antibody‐based ELISA assay and the mass spectrometry based PRM assay (*r* = 0.907, *p* (two tailed) < 0.0001, 95% confidence interval 0.8603–0.9384).

We further confirmed the CRP measured with PRM‐MS by comparing it to CRP measured as part of the standard of care for IBD patients using an established antibody‐based clinical assay. CRP is frequently measured as part of the diagnostic and monitoring procedures for IBD patients and was independently measured in 91 IBD patients at the same time point of sample collection (42 at 8 weeks, 44 at 16 weeks, and 5 at 24 weeks) in a clinical lab using standard procedures.^[^ ^45^ ^]^ While not all peptides could be validated due to the availability of similar clinical tests, we examined the correlation between the CRP level measured by PRM and the clinically measured CRP level. We observed a strong correlation between CRP levels measured by MS and a standard clinical test, with a Spearman correlation of 0.907 (Figure 9).

We also performed preliminary analysis on the protein differential expression of the HSP between the IBD and control subjects. Although the current biomarker panel was not specifically designed to identify biomarkers for IBD, we observed that several proteins, including IGHA1, ANT3, and A2MG, were significantly differentially expressed between the two groups (Table S8). More importantly, we observed significantly lower albumin level (unadjusted *P* = 5.08E^−8^) and higher CRP levels (unadjusted *P* = 0.0001) in IBD cases. These preliminary findings need to be verified in additional cohorts, and although the underlying biological significance is beyond the scope of current study, once replicated, those proteins could potentially inform an expanded biomarker panel aimed at improving risk stratification and might shed light on the underlying pathogenesis of IBD.

It is worthwhile to note that using the Stellar platform, we observed pronounced differences for CRP and albumin between IBD and control groups. This mirrors their well‐established roles as indicators of systemic inflammation and IBD disease burden. CRP rises within hours of IL‐6‐driven inflammatory signaling and correlates with mucosal ulceration, transmural activity, and endoscopic indices, whereas hypoalbuminemia reflects chronic inflammation and protein‐losing enteropathy and predicts poorer therapeutic response and postoperative outcomes. Moreover, the consistency of Stellar‐derived CRP with clinical measurements further highlights the platform for biomarker identification and validation.

## Conclusion

The final multiplex PRM assay offers enhanced sensitivity without compromising throughput, making it a powerful tool for the accurate quantification of multiple biomarkers and translational research, including IBD research, enabling the identification and quantification of a panel of biomarkers with high precision. Future work will focus on further refining the assay for improved performance and broader clinical applications, including other inflammatory and chronic diseases. Additionally, the scalability of this platform offers opportunities for longitudinal studies and real‐time monitoring of disease progression, paving the way for precision medicine approaches in clinical proteomics. This work will establish a robust framework for precision and scalability in biomarker validation and could be broadly applicable to other translational studies and disease contexts requiring precise relative quantification of extensive protein sets. Overall, these results validate the feasibility of the multiplex PRM assay for high‐throughput proteomic analyses, with robust reproducibility, stability, and performance across a large clinical cohort.

## Conflict of Interests

The authors declare no conflict of interest.

## Supporting information



Supporting Information

Supporting Information

## Data Availability

The mass spectrometry proteomics raw data files have been deposited to the The ProteomeXchange ID: PXD062364.
